# The winter holidays are glorious—except when they’re not

**DOI:** 10.1038/s44319-024-00318-z

**Published:** 2024-11-11

**Authors:** Shina Caroline Lynn Kamerlin

**Affiliations:** Georgia Institute of Technology, School of Chemistry and Biochemistry, 901 Atlantic Drive NW, Atlanta, GA 30332-0400 USA

**Keywords:** Careers

## Abstract

The winter holidays are a time to celebrate, but can be oppressive for those who cannot. It is therefore important to look after trainees and colleagues who miss their loved ones on Christmas day.

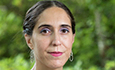

As I write this column, it is early October. I live in a neighborhood where people take Halloween very seriously, and the decorations are already up on every street. Soon we will move from Halloween to Thanksgiving and to Christmas along with other winter holidays. Towns will be filled with beautiful lights, Christmas markets will open (even in Atlanta!) and we will enjoy all the wonderful food and gifts and Holiday parties that remind me of my childhood in Europe.

For most of us, the winter holidays are a wonderful time of closeness with friends and family, a time to celebrate together and break the darkness with light; many cultures and religions have similar festivals of light at around the same time of year. But not everyone is sharing in our joy. It can be debated whether suicide rates really go up around the Holidays: there is data that suggests that this is a myth. Other data, however, shows that, at least in Sweden, suicides are not significantly increased on Christmas and New Year’s Eve, but spike on New Year’s day (Hadlaczky and Hökby, 2018). Without extending to as severe outcomes as suicide, it’s clear that the Holidays can be a very challenging time from a mental-health perspective. Holiday depression is of course likely to be exacerbated if one already has underlying mental health issues, and suddenly is left alone while everyone else is celebrating. This can be particularly challenging in parts of Europe where Christmas is still very much a family affair, which is great if you have family to celebrate with, but if you do not, you can be so incredibly alone. When I was young, you would see nobody out on the streets in Sweden on Christmas eve after noon as everyone was indoors with their families. Everything was quiet in the winter darkness. That loneliness can be extremely oppressive if you miss your family and friends.

In this context, as the Holiday season approaches, it is important for those of us who have responsibility for trainees—not just faculty but also postdocs and staff scientists—to remember two important things: even without additional challenges of loneliness on Holidays, mental health problems are, unfortunately, rife among undergraduate students, PhD students and postdocs; and the global nature of science means that many of our trainees are far from home, with their normal circle of friends, family and sometimes even partners elsewhere in other countries or even continents. The sense of missing them will be amplified even further at a time when everyone else is enjoying time with family or friends. This can also be true of faculty, who might be far away from home. When combining the well-known phenomenon of holiday depression, with the broader mental health issues faced by students (for example, a 2015 survey by the National Union of Students suggested that the majority of UK students face mental health issues), this makes the Christmas holidays a particularly vulnerable period for the mental health of PhD students. On this note, for anyone reading this who may need support, King’s College London provide helpful advice on maintaining mental health during the Holiday season.

Thus, as you prepare to celebrate, keep an extra eye out for your colleagues and the members of your research team. Pre-pandemic, I had a tradition of inviting all members of my research group who were still in Uppsala over the holidays to my home on Christmas day for pudding and a small get-together. Many went on to their own celebrations afterwards, but that way I knew whether they had friends or not, that no one needed to be alone on Christmas day. This tradition fell by the wayside during the pandemic, but writing this piece has reminded me of how important it is to start it again. You might find other solutions to look out for your teams, but whatever it is, please don’t forget to do so. As the title of this piece states, the winter holidays are glorious, except when they’re not. And when they’re not, they can be a truly oppressive time of the year.

## Supplementary information

Peer Review File
