# Supplementary material for: The winter holidays are glorious—except when they’re not
Source: EMBO Rep. 2024 Nov 11;25(12):5223–4. doi: 10.1038/s44319-024-00318-z (PMC11624255; doi:10.1038/s44319-024-00318-z)
Supplement: Supplementary file 1 — Peer Review File [file 44319_2024_318_MOESM1_ESM.pdf]

EMBO reports

The Winter Holidays Are Glorious - Except When They're Not

Shina Caroline Lynn Kamerlin

Corresponding author(s): Shina Caroline Lynn Kamerlin (skamerlin3@gatech.edu)

Review Timeline:

Submission Date:	21st Oct 24
Accepted:	4th Nov 24

Editor: Holger Breithaupt

Transaction Report: This article type is not peer reviewed.
